# Odour-Evoked Memory in Dogs: Do Odours Help to Retrieve Memories of Food Location?

**DOI:** 10.3390/ani10081249

**Published:** 2020-07-23

**Authors:** Angelo Quaranta, Serenella d’Ingeo, Marcello Siniscalchi

**Affiliations:** Department of Veterinary Medicine, Section of Animal Physiology and Behaviour, University of Bari “Aldo Moro”, 70121 Bari, Italy; angelo.quaranta@uniba.it (A.Q.); serenella.dingeo@uniba.it (S.d.)

**Keywords:** dog, olfaction, odor-evoked memory, cognition, physiology, behaviour

## Abstract

**Simple Summary:**

The ability of odors to evoke past memories has been widely reported in humans. Although olfaction is generally considered as the most important sense in dogs, little is known about its relationship with memory in this species. To investigate this issue, we trained dogs to memorize the location of five rewards while a specific odor (i.e., vanilla) was dispersed in the environment. After 24 h delay, dogs were divided in three groups, which performed two trials of the same spatial task in different conditions. The first group received a control odor (i.e., apple) in the first trial and the vanilla odor in the second trial; vice versa, the second group was exposed to the vanilla odor in the first test and to the apple odor in the second one. The third group, instead, performed the tests with no odors. We found that the exposure to vanilla odor significantly improved dogs’ performance in the spatial task, suggesting that the odor aided dogs to recall specific and detailed memories originally formed in its presence.

**Abstract:**

The ability of odors to spontaneously trigger specific memories has been widely demonstrated in humans. Although increasing evidence support the role of olfaction on dogs’ emotions and cognitive processes, very little research has been conducted on its relationship with memory in this species. The present study aimed at investigating the role of olfaction in the recall of detailed memories originally formed in the presence of a specific odor (i.e., vanilla). To test this, three groups of participants were trained with the same spatial learning task while a specific odor (i.e., vanilla) was dispersed in the testing room. Subjects were then divided in three experimental groups and after 24 h delay, they were presented with the same spatial task. The first group (Group 1) performed the task in the presence of a novel odor (i.e., control), whereas the second (Group 2) and the third group (Group 3) carried out the test in the presence of the vanilla odor and no odor (Group 3), respectively. After a brief delay, the test was presented again to the three groups of dogs: subjects of Group 1 were now tested in the presence of the vanilla odor, whereas the Group 2 was tested with the control odor. The Group 3 received no odor in both tests. A significant improvement of dogs’ performance was registered in the control-vanilla odors condition (Group 1), suggesting that the exposure to the odor presented at the encoding time would prompt the recall of spatial memories in dogs.

## 1. Introduction

Dog olfactory abilities are widely recognized [1] and have been extensively used by humans for social (e.g., the detection of explosive or narcotics [2]) and even medical purposes (e.g., for the diagnosis of several human diseases [3,4]) during the last decades. Although it is generally considered as the most important sense in dogs, to date very little research has investigated the role of olfaction in these species. Recent literature shows that dogs distinguish other individuals, their sex and reproductive state through their smell [5,6] and perceive social information conveyed by odors, like conspecific and human emotions [7]. The exposure to olfactory emotional signals [8] and to different types of essential oils [9,10] influences dog behavior and, as recently reported by Duranton and Horowitz [11], a regular olfactory-based activity (i.e., nosework) improves dogs’ affective state and welfare. Furthermore, a relationship between olfaction and cognition has been described in dogs. In particular, it has been shown that dogs form an olfactory search image for explosive odors [12] and represent the object they are searching for, showing signs of surprise when finding an object different to the one expected [13]. Overall, results of these experiments provide clear evidence about the significant involvement of the olfaction on dogs’ emotions and cognitive processes. Nevertheless, further studies are still needed to address this issue and to deepen our knowledge in such field.

On the other hand, the link between olfaction and cognition has been extensively investigated in humans. Olfactory cues influence not only human thinking, actions and decisions in a non-conscious manner [14,15] but also human judgment [16] and behavior toward other people (e.g., clean scent increases helping behavior [17]). Recent literature shows also that odors affect human mood [18] and working performance when they are previously associated with the emotion of frustration [19].

One of the most interesting aspects of the relationship between olfaction and cognition in humans is the ability of odors to spontaneously trigger specific memories, a process widely known as Proust phenomenon. Marcel Proust, indeed, was the first who described the power of odor to recall a subject’s past events in *A la Récherche du Temps perdu*, reporting how the smell of a madelèine biscuit soaked in tea brought him back to a childhood experience [20]. A thorough scientific investigation of the existence of such phenomenon has been performed in the last twenty years [21]. It has been shown that odor-evoked memories are experienced as more emotional and evocative (i.e., the feeling of being brought back in time) than memories elicited by other sensory cues (i.e., visual and auditory cues) [22,23,24,25]. The relationship between memory, olfaction and emotions is further supported by neurophysiological studies, which demonstrate a stronger activation of the hippocampus and the brain areas related to the emotional processing, such as for people the amygdala and temporal regions [26,27], and a higher heart rate [28] during the recall evoked by odors than by visual or musical stimuli, respectively. The power of odors to retrieve emotional memories has an important clinical implication for several mental disorders, as for people with posttraumatic stress disorder (PTSD). It has been shown that trauma-related smells are strong triggers of traumatic memories in PTSD patients and help them to recall more details of the traumatic event [29]. However, the ability of odors to evoke richer and more accurate memories is still debated. Although few studies found that odors do not elicit more detailed memories than other sensory cues [28,30], there is an increasing evidence that the amount of details recalled by odors related to the experienced event is significantly higher than the one evoked by visual or verbal cues [31,32,33,34]. These findings pointing to the possibility that an odor become strongly tied to a specific event, whose memory is than “unlocked” only by that stimulus [21].

Given the remarkable olfactory ability of dogs, the present study aimed at investigating the relationship between olfaction and a specific aspect of cognition in this species, namely memory. In particular, we analyzed whether odor cues can aid the recall of specific and detailed memories originally formed in the presence of that odor, as previously described for human beings. 

## 2. Materials and Methods 

A laboratory-based approach was used [35], in which subjects were presented with a spatial learning task. Considering that dogs have been shown to search accurately for visibly displaced objects and retain the memory of their location until 4 min [36,37,38,39], in our experiment, and in particular in the encoding phase, five rewards were visibly hidden by an experimenter under five out of 29 plastic cups located in different places in an empty room. Since spatial learning in dogs (i.e., the ability to locate the position of an object in the space) is based upon both egocentric and allocentric cues, that is the position of the subject and of an external referent or landmark respectively [40], a unique item was placed next to each plastic cup [41] as an allocentric cue. Our pilot tests confirmed the hypothesis that dogs’ memory capacity in a spatial task was very low [42]. Therefore, we decided to use the same landmark (i.e., a red plastic container) as an allocentric cue for the five rewards during the training phase in order to facilitate dogs’ memorization of its position. Moreover, since previous studies showed that age impairs spatial learning in dogs [41,43], our population was made up of subjects whose age range between 7 months and 8 years. The positioning of the rewards in the encoding phase occurred in the presence of an odor (i.e., vanilla), which served as the potential trigger during the subsequent phase, the memory retrieval phase, when subjects had to recall spontaneously the place where the reward was hidden. It was predicted that the vanilla odor would prompt the recall of the location of the rewards, and, as a consequence, it would significantly affect dogs’ performance in the spatial task.

### 2.1. Participants

Eighteen domestic dogs of various size and breeds were involved in the study. Participants were four males (one neutered) and 14 females (seven spayed), whose age range between 7 months and 8 years (2.83 ± 2.17; mean ± SD). All dogs were pets living in households. Before the experiment begun, clinical and behavioral evaluations were performed on all the sample by two veterinarians of the Department of Veterinary Medicine, University of Bari. None of the tested dogs suffered from any organic or behavioral diseases.

### 2.2. Experimental Apparatus

**Figure 1:**
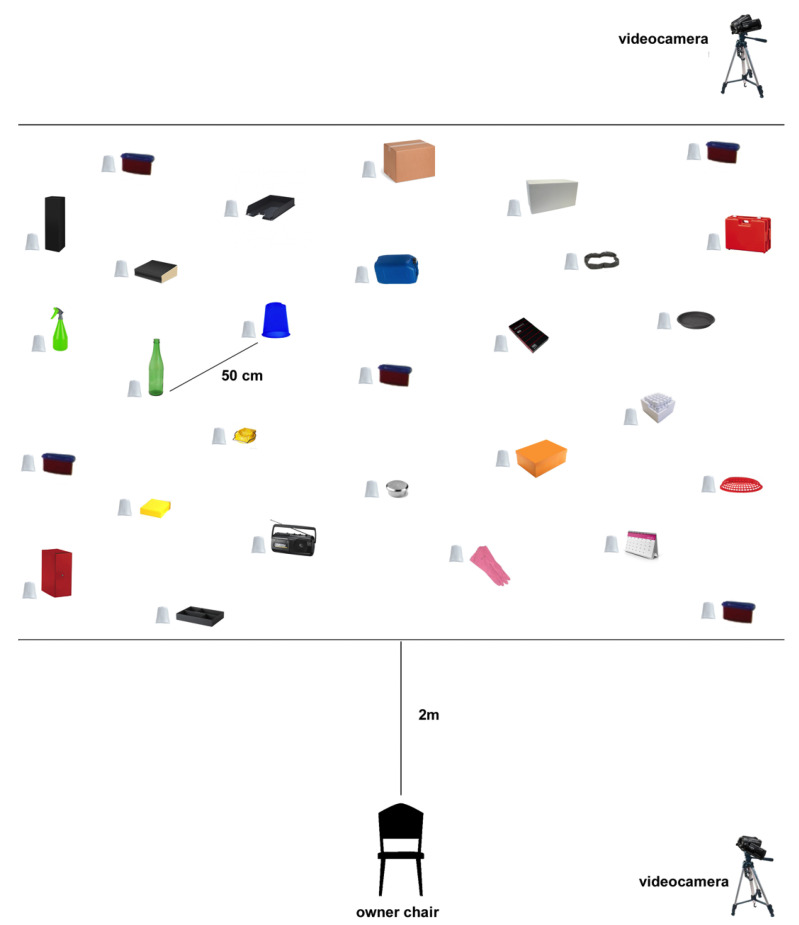
Testing setup.

The experiment was carried out in an isolated room of the Department of Veterinary Medicine, University of Bari. Twenty nine plastic cups were positioned on the floor (Figure 1). Under each cup, a single ramekin was placed, where an experimenter left the rewards (i.e., a slice of würstel) in the encoding phase. It was employed to avoid that food used as reward would leave any odour traces on the floor. This prevent dogs to follow food odors in the memory retrieval test, where dogs’ ability to recall information about food location learned in the encoding phase was analysed (no food was present in this phase). A unique item was located next to each plastic cup as an allocentric cue, so that a total of 29 items were present in the room. They were all different aside from the 5 that were associated with the reward (i.e. red plastic containers). To obtain the rewards, dogs had to take the cup off using their paw or muzzle. Therefore, the plastic cups were fastened with tape on the floor only on one side. 

A chair, where the owner sat during the test, was centrally position in the room, facing the items and at a distance of 2 m from them. The odours were dispersed through an automatic pump-driven dispenser (Freshmatic^®^ automatic spray, Air Wick^®^, Reckitt Benckiser, Slough, UK). Olfactory stimuli used in the experiment were vanilla (Air Wick^®^ Vanilla Passion^®^, Reckitt Benckiser, Slough, UK) and apple (Air Wick^®^ Winter Apple^®^, Reckitt Benckiser, Slough, UK). 

Two full HD digital video-cameras (Sony Alpha 7 II ILCE-7M2K^®^, Sony Corporation, Tokyo, Japan) were used to register dogs’ behaviour during the experiments. They were positioned on a tripod at the two right corners of the room, focusing on the testing area.

### 2.3. Procedure

The experiment was carried out in two phases, performed in sequence. In each phase, dogs were tested individually.

#### 2.3.1. Phase 1: Memory Encoding

In phase 1, subjects had to memorize the reward location after observing an experimenter positioning them, in the presence of the vanilla odour. Before the test begun, each dog was led in the room by its owner and was allowed to freely explore the testing area and the apparatus for about 5 min (no food was present). The dog and its owner left then the room and the experiment started. During the encoding phase, the vanilla odour was dispersed in the testing area using the above-described device. Owners led their dogs in the room and sat on the chair, where they remain for the test duration, holding the dogs on the leash. An experimenter (the same for all the subjects) positioned the rewards in the ramekin under the 5 plastic cups placed next to the red plastic containers, while catching the dog’s attention (by calling its name or producing a little sound). To position each reward, the experimenter opened the cup, left the reward inside the ramekin and then closed the cup. The order of the rewards positioning was the same for each trial. When all the rewards were placed, the experimenter positioned himself behind the owner and stood still until the trial ended. Once she reached this location, the owner let the dog off the leash, who freely explore the cups to find the hidden rewards. To obtain the food, dogs had to tip the cup over with their paw or their muzzle. Every time the subject found the food, it was verbally rewarded by its owner (“Bravo!”) in order to strengthen the positive valence of this reinforcement. The trial lasted until all the rewards were found. The owner and the dog left then the room. Soon after the end of each trial, the floor of the testing area was washed with baking soda and all the ramekin and cups that were touched by the dog during the test were removed and substituted with new ones. This prevented dogs using their own scents marks to find the rewards in the subsequent trial. This procedure was carried out until the dogs found all the rewards consecutively, with no errors (i.e., searching for them under wrong cups), within 2 min and for two consecutive trials. All dogs completed the training phase in about one hour.

#### 2.3.2. Phase 2: Memory Retrieval

In the phase 2 dogs had to spontaneously recall the location of the rewards and to explore the cups where they were previously placed. This phase occurred in the presence of the vanilla odour, a control odour (apple) or no odour. The procedure followed the methods described by Aggelton and Waskett [33] to study odour-evoked memory in humans.

Subjects were assigned to one of three experimental groups (6 in each group), which were age balanced. They were tested twice with the same procedure but under different conditions, in the presence of:

Group 1: control odour (i.e., apple)—vanilla odour;
Group 2: vanilla odour—control odour;
Group 3: no odour—no odour.

Phase 2 took place after 24 h from the phase 1. In each trial, dogs were led by their owner on the leash in the testing room and were unleashed as soon as the owner sat on the chair. The experimenter stood behind him avoiding any interactions with dogs. Since the odour of the rewards could influence dogs’ searching behaviour, in this phase no food was placed near the rewarded items. Thus, dogs’ behaviour was guided exclusively by the memories formed in the encoding phase. In addition, to avoid any influence on the trial repetition, no vocal rewards were used by the owners when subjects explored the correct items (i.e., the rewarded ones). Dogs’ spontaneous searching behaviour was recorded during the trials. Each trial lasted for a maximum of 4 min but, if dogs explored all the correct five items before the given time limit, the trial was interrupted. After an interval of 30 min, which was necessary to remove the odours dispersed in the room and to wash the floor, the procedure was repeated. The only difference was that the trial was carried out in the presence of a different odour for the Group 1 and 2. Regarded the Group 3, instead, participants performed both of the trials with no odour in the testing area.

### 2.4. Data Analysis

The recorded videos were analysed by two trained experimenters. The following parameters were considered:

Total time: The total time (s) spent by each subject to explore the five correct items (i.e., from the moment in which it was unleashed until the dog touch with its muzzle or paw the last correct item). A score was allocated for each of the following behaviour:

2: dogs touched with their muzzle or paw a correct item;
1: dogs made an olfactory check to explore a correct item;
0: dogs explore again the same item (for both the correct and incorrect ones);
−1: dogs made an olfactory check to explore an incorrect item;
−2: dogs touched with their muzzle or paw an incorrect item.

### 2.5. Ethics Statement

The experiments were carried out according to the protocols approved by the Italian Minister for Scientific Research in accordance with EC regulations and were approved by the Department of Veterinary Medicine (University of Bari) Ethics Committee EC (Approval Number: 20-2020). In addition, before the experiment began, informed consent was obtained from all the participants included in the study.

## 3. Results

### 3.1. Total Time

**Figure 2:**
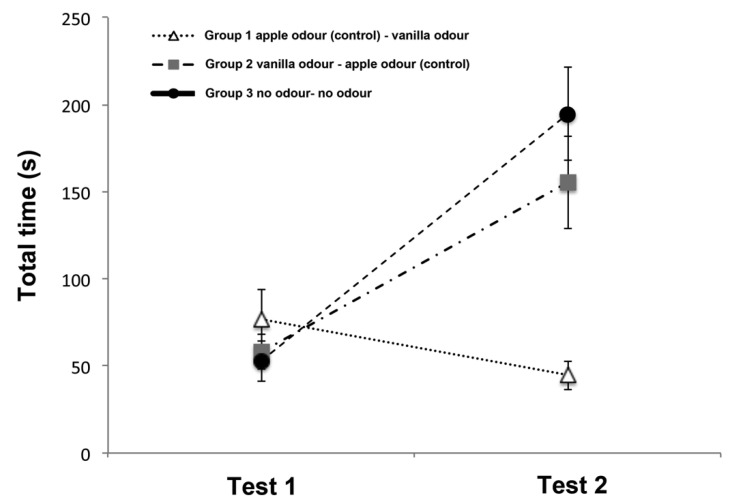
Graph showing the mean total time of the three groups spent exploring the correct 5 items on Test 1 and Test 2 in the Memory Retrieval Phase. The vertical lines show the standard error of each mean.

Results for total time spent exploring the correct 5 items are shown in Figure 2. The analysis of variance revealed a significant main effect of trials (ANOVA: F(1,15) = 27.781, *p* < 0.001), odors (F(2,15) = 15.888, *p* = 0.020) and trials × odors interaction (F(2,15) = 15.888, *p* < 0.001). Overall there was a significant increase over the two trials in the time spent to find the correct items (first trial = 62.65 ± 7.731; second trial = 131.54 ± 12.83; mean ± SD; post-hoc analysis Fisher’s protected LSD: *p* < 0.01). However, interaction analysis revealed that this trend was shown only for the Group 2 (first trial (58.19 ± 9.95; mean ± SD) vs. second trial (155.49 ± 26.70; mean ± SD) (t(5) = −4.070, *p* = 0.010)) and the Group 3 (first trial (52.94 ± 11.69; mean ± SD) vs. second trial (194.63 ± 26.51; mean ± SD) (t(5) = −6.373, *p* = 0.001)) but not for the Group 1 (first trial (76.79 ± 17.37; mean ± SD) vs. second trial (44.49 ± 8.04; mean ± SD) (t(5) = 1.478, *p* = 0.199).

No other statistically significant effects were observed: age (F(5,12) = 1.363, *p* = 0.305) and trials × age interaction (F(5,12) = 2.565, *p* = 0.084); sex (F(1,16) = 1.173, *p* = 0.295) and trials × sex interaction (F(1,16) = 0.002, *p* = 0.967).

### 3.2. Score

**Figure 3:**
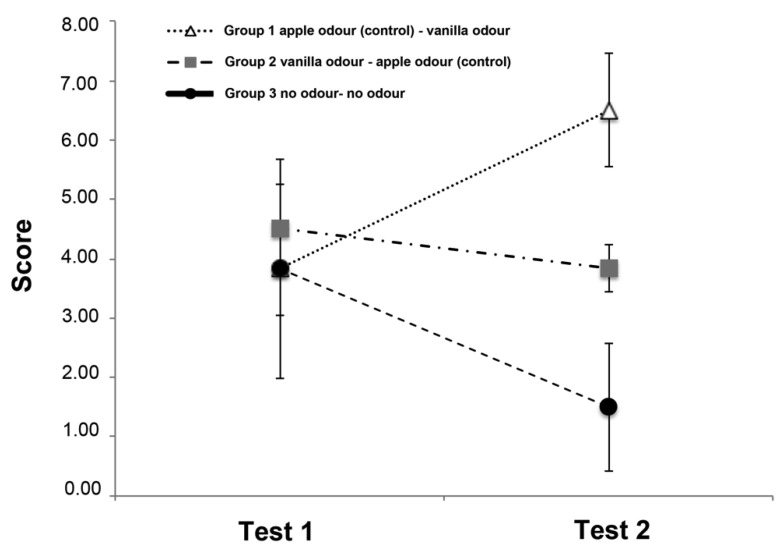
Graph showing the mean scores of the three groups on Test 1 and Test 2 in the Memory Retrival Phase. The vertical lines show the standard error of each mean.

The score of the three groups is shown in Figure 3. No effects of both odour (F(2,15) = 1.290, *p* = 0.304) and trials (F(1,15) = 0.023, *p* = 0.883) were found. A statistical significant odour × trial interaction was observed (F(2,15) = 3.941, *p* = 0.042), which indicates a significant improvement in the dogs’ performance in the second trial for the Group 1 (first trial (3.83 ± 0.79; mean ± SD) vs. second trial (6.5 ± 0.95; mean ± SD) (t(5) = −6.325, *p* = 0.001)) but not for the others two groups (Group 2: first trial (4.5 ± 0.76; mean ± SD) vs. second trial (3.83 ± 0.40; mean ± SD) (t(5) = 1.195, *p* = 0.286; Group 3: first trial (3.83 ± 1.85; mean ± SD) vs. second trial (1.50 ± 2.02; mean ± SD) (t(5) = 1.107, *p* = 0.319). Although, as would be expected, during test 1 Group 2 has a higher absolute value score than the other two groups, this value does not reach statistical significance.

No other statistically significant effects were observed: age (F(5,12) = 0.188, *p* = 0.962) and trials × age interaction (F(5,12) = 1.180, *p* = 0.375); sex (F(1,16) = 0.662, *p* = 0.428) and trials × sex interaction (F(1,16) = 0.293, *p* = 0.596). 

## 4. Discussion

Here we report for the first time the effectiveness of odors as contextual retrieval cues for spatial memory in dogs. The results showed that the exposure to the odor (i.e., vanilla) that has been present during the memory formation, improved the recall of spatial memories about the location of rewards in a spatial task. In addition, subjects exposed to the training odor during the recall test didn’t show an increase in the time spent exploring and opening the correct items with respect to the training period suggesting the maintenance of attention levels. These findings are consistent with the encoding specificity principle [44] which states that “memory for material is enhanced when contextual stimuli encoded along with the target information are present at retrieval” [45]. In our study, which relied on an associative learning procedure [46], an ambient odor (i.e., vanilla) was present during the learning process for a set of “to-be-remembered items” and the later recall phase, within the same experimental setup. We found that the exposure to the vanilla odor in the memory retrieval phase significantly affected dogs’ performance in the same spatial task carried out 24 h after the training. Specifically, when the vanilla odor was dispersed in the testing room, dogs spent less time and made fewer mistakes (i.e., short total time time and high scores) in visiting the five items that had been associated with the food reward during the encoding phase. Since the vanilla odor appeared to be an effective contextual memory cue, our results suggest the existence of a specific type of odor-evoked memory in dogs, namely context-dependent memory. According to Tulving [44], in the context-dependent memory the environmental features encoded in the memory could facilitate the recall of such memory when subsequently encountered [45]. We found that the environmental odor that was present during the memory formation and retrieval process significantly improve the recall of the location of the rewards previously learned. This suggests that the odor has been part of the encoding environment and has been stored as a salient cues that strongly tied with the spatial information learned in the experimental context. In a previous study, Aggleton and Waskett [33] reported that human participants exposed to the odors dispersed in a museum (Jorvik Viking Centre, York, UK) recalled more details about the exhibition than people exposed to a different (control) set of odors or no odors. Similarly, in the present study three different experimental conditions were considered. Dogs were divided in three groups, which performed two trials of the same spatial task in different conditions. The first group received a control odor (i.e., apple) in the first trial and the vanilla odor in the second trial; vice versa, the second group was exposed to the vanilla odor in the first test and to the apple odor in the second one. The third group, instead, performed the tests with no odors. The effectiveness of the vanilla odor in the recall of the memory about the food locations was clearly observed in the control-vanilla condition, namely in the second trial of the memory retrieval phase. Dogs’ performances in the first trial, instead, were found to be similar in all the three different conditions (i.e., apple, vanilla and no odor). The lack of significant differences in the three odor-contexts of the first trial could be linked to dogs’ high expectation to receive food as in the encoding phase, which took place only 24 h before. In other words, it could be possible that dogs’ high expectation to find the reward in the first trial could have mitigated the influence of the odor cue on subjects’ performances. Both the time needed to visit the rewarded items and the score, indeed, were similar in the three groups. However, the score of the second group, which carried out the test in the presence of the vanilla odor, was higher than in the other groups (although it does not reach statistical significance), suggesting a certain tendency of dogs to make fewer mistakes in searching for the rewards when exposed to vanilla odor in the first trial. Therefore, it is possible that the limited population size considered in this study could have affected the observable differences between the groups. Future studies involving a larger sample population are needed to better clarify the nature of these findings. 

On the contrary, the effectiveness of the vanilla odor for the memory recall was evident in the second trial, where we registered a general decline of dogs’ performances, as demonstrated by the longer time spent to visit the rewarded items and the lower scored obtained. These findings might be due to the incentive changes phenomenon (no reward in the first trial compared to the encoding phase) that could have markedly decreased subjects’ motivation and expectancy to obtain the reward in the second trial [47]. However, dogs that were exposed to the vanilla odor in the second trial performed significantly better in terms of time and score, suggesting that the vanilla odor cued the memory about the food location formed in the training phase. Although this evidence do not preclude the possibility that the increase of dogs’ performance was due to a general higher motivation to receive the rewards cued by the vanilla odor (after an un-rewarded trial), this seems to be unlikely. The high score registered as well as the short time needed to visit the five correct items suggests that dogs performed the task making very few mistakes that could not be explained by a general activation of the motivation of hunger and a non-specific searching-for-food behavior. On the contrary, our results suggest that the vanilla odor facilitated the recall of the specific spatial information about the food location, leading dogs to spend short time to visit all the correct items and to make few mistakes (high scores). However, we cannot entirely rule out the possibility that our results reflects the incentive contrast phenomenon, which could explain the dogs’ higher performance observed in the second trial as an effect of an increased motivation and expectancy to receive food after an un-rewarded trial (the first trial). The influence of incentive changes and of the positive expectation for receiving food rewards (e.g., leaving the rewards’ scents on all the cups in the retrieval phase) on the recall of spatial memory in dogs are important issues that need to be further investigated.

An interesting aspect of the odor-evoked memory concerns its relationship with emotions. It has been shown that a heightened and salient emotional state experienced during the encoding of information associated to a specific odor would enhance the ability of such odor to retrieve the information learned [48]. Therefore, the significant improvement in dogs’ performance could be further explained by the high and positive affective state experienced in the training/encoding phase, which was due to the presence and the obtaining of the rewards. Nevertheless, future experiments are needed to verify whether changes in subjects’ emotional state, in absence of motivation of hunger, could alter the effectiveness of odors as retrieval cue.

The existence of a unique association between an odor and the spatial information (i.e., reward location) created during the encoding phase is further supported by the relationship between olfaction and the neural structures involved in several cognitive processes, including memory, emotions, and associative learning [21]. In particular, exclusive anatomical connections between the olfactory cortex and the hippocampus suggest that this neural circuit could be the main involved during contextually odor cued spatial memory recall tests [49,50]. The hippocampus in fact, is crucial for spatiotemporal context representation and its association with the sensory details, which come from the environment to form episodic memories [50,51]. Furthermore, recent studies in behaving rats have also suggested a fundamental role of the piriform cortex in modulating the functioning of the neural network between the olfaction and the hippocampus. Specifically, the work of Strauch and Manahan-Vaughan [49] provides evidence that activity in the piriform cortex evokes field potentials in the dentatus gyrus supporting the hypothesis about the main role of the piriform cortex in specific control of hippocampal information processing and encoding. The piriform cortex represents the largest domain of the olfactory cortex playing a pivotal role of olfactory information processing because its neural network connections with all parts of the lateral entorhinal cortex [52,53]. The latter drives unimodal sensory information along with attentional and motivational information conveyed by the anterior brain regions to the hippocampus. On the other hand, behavioral, anatomical and electrophysiological evidences support a role for the dentate gyrus, which appears to be largely sensitive to olfactory stimuli, in mnemonic processing of spatial information [49,54].

Overall, our results support the findings of human studies demonstrating the ability of odor to trigger memories of specific episodes [31,32,33,34]. These memories become strongly tied with the odor that was present at the moment of the memory encoding [21]. In fact, we observed that different but not specific odors failed to aid dogs to retrieve more details about the location of food. Therefore, our findings suggest that memories elicited by odors are markedly accurate and specific.

Furthermore, our findings raise important issue about the ecology of the canine species. Spatial memory, indeed, is fundamental for learning the position of object in the space to recognize its own territory [40,55] but also to remember the location of food sources. In fact, our results demonstrate that odors represent a crucial cue for facilitating the recall of memories about food location, and attribute, therefore, to olfaction a key role for individual survival. They also might have practical implications for the treatment of fear- or phobia-related behavioral problem of dogs. Since it has been shown that trauma-related smells contribute to recall the traumatic event in humans [29], the odors associated with the event eliciting the emotion of fear in dogs could be used as a stimulus in the most common techniques that treat phobia disorders in this species (i.e., desensitization or counterconditioning). However, the link between olfaction, memory and emotion merits further consideration.

## 5. Conclusions

Overall, our results indicated the existence of a relationship between olfaction and memory in dogs, supporting previous evidence of human studies about the crucial role of odor cues to retrieve specific and detailed memories formed in the present of that odor. This raises important issue about the role of olfaction in several cognitive processes in dogs, including emotional memories, as well as in the ecology of the canine species, which still need to be address. Future studies using different experimental paradigms on a larger sample population are required to deepen and strengthen knowledge of this original and currently little investigated research field.
